# How to Treat Today? Oral and Facial Cancer-Associated Venous Thromboembolism

**DOI:** 10.3390/ph16071011

**Published:** 2023-07-17

**Authors:** Mária Janíčková, Tomáš Bolek, Lucia Stančiaková, Norbert Nagy, Marián Mokáň, Matej Samoš

**Affiliations:** 1 Department of Stomatology and Maxillofacial Surgery, Jessenius Faculty of Medicine in Martin, Comenius University in Bratislava, 036 59 Martin, Slovakia; maria.janickova@uniba.sk; 2 Department of Internal Medicine I, Jessenius Faculty of Medicine in Martin, Comenius University in Bratislava, 036 59 Martin, Slovakia; ato.bolek@gmail.com (T.B.); norbi.nagy.nn@gmail.com (N.N.); mokanmarian@gmail.com (M.M.); 3 National Centre of Hemostasis and Thrombosis, Department of Hematology and Blood, Transfusion, Jessenius Faculty of Medicine in Martin, Comenius University in Bratislava, 036 59 Martin, Slovakia; lstanciakova@gmail.com; 4 Division of Acute and Interventional Cardiology, Department of Cardiology and Angiology II, Mid-Slovakian Institute of Heart and Vessel Diseases (SÚSCCH, a.s.) in Banská Bystrica, 974 01 Banská Bystrica, Slovakia

**Keywords:** oral and facial cancer, cancer-associated venous thromboembolism, direct oral anticoagulants, parenteral anticoagulation

## Abstract

The exact incidence of cancer-associated venous thromboembolism (CA-VTE) in patients with oral and facial cancer (OFC) is not exactly known, and this risk is empirically considered to be low. However, this suggestion may result in disease underdiagnosis, prolong the initiation of adequate therapy, and consecutively increase CA-VTE-related morbidity and mortality. In addition, there might be specific clinical problems in the treatment of CA-VTE in patients with oral and facial cancer, such as swallowing difficulties, that might limit the possibilities of oral anticoagulation. Finally, there are limited data regarding the optimal treatment of CA-VTE in patients with oral and facial cancer, and this includes data on novel therapeutic strategies, including the use of direct oral anticoagulants. This article reviews current data on the optimal treatment strategy for CA-VTE in patients with OFC.

## 1. Introduction

The risk of venous thromboembolism (VTE) is significantly higher in patients with active cancer (about 4-fold generally and about 7-fold in those receiving cytostatic or surgical treatment), and VTE is a leading cause of death in patients with active cancer [1,2]. Furthermore, the treatment of cancer-associated VTE (CA-VTE) might be challenging due to several factors, such as cancer-related worsening of the patient’s general condition (malignant cachexia), a higher risk of recurrent VTE and bleeding, and the possible interaction of anticoagulant drugs with anticancer therapies [3].

The exact incidence of CA-VTE in patients with oral and facial cancer (OFC), most frequently represented by oral squamous cell carcinoma, is not exactly known. Nevertheless, the risk of CA-VTE in these patients is generally (empirically) considered to be low. However, this suggestion may result in disease underdiagnosis, prolong the initiation of adequate therapy, and consecutively increase CA-VTE-related morbidity and mortality. In addition, there might be specific clinical problems with the treatment of CA-VTE in patients with OFC. For example, oral intake of the drug might be problematic due to impaired swallowing in patients undergoing significant tumor resections in the oral and facial areas or in those treated with radiation therapy [4]. Finally, there are limited data regarding the optimal treatment of CA-VTE in patients with OFC, and this also includes data on novel therapeutic strategies, including the use of direct oral anticoagulants (DOACs). The aim of this article is to provide up-to-date data on the optimal treatment strategy for CA-VTE in patients with oral and facial malignancies.

## 2. Methods

This article is designed as a narrative (non-systematic) review article. To fulfill our aim, we performed a search of the three most important medical literature databases—Web of Science, PubMed Central, and Scopus—using topic-related keywords as follows: “cancer-associated venous thromboembolism” or “cancer” + “deep venous thrombosis” or “pulmonary embolism” and “oral” and “facial cancer” or “oral squamous cell carcinoma” and “treatment” or “therapy” to identify relevant sources from the currently available medical literature. Subsequently, search results were checked and reviewed by the authors, and a narrative review article summarizing the most important data from relevant articles was written.

## 3. CA-VTE in Patients with OFC

Generally, malignant cells can activate coagulation directly by producing procoagulant substances such as tissue factor (TFs) or pro-coagulant microparticles. As for oral cancer, the tissue factor can be considered the most important procoagulant protein [5,6,7].

In addition, microparticles from oral cancer cell lines could induce tissue factor synthesis and platelet aggregation [8]. In vitro cancer models have proven the role of microparticles in thrombus formation. High concentrations of circulating microparticles have been reported in patients with oral cancer [9].

Additionally, an inflammatory response often seen in patients with OFC may contribute to the increased risk of thromboembolism [10]. For example, elevated levels of pro-coagulable (thromboxane A2, von Willebrand factor, and prostacyclin) proteins and decreased levels of thrombo-protective thrombomodulin plasma levels have been reported in patients with oral cancer [11,12]. Tumor-associated inflammation plays a crucial role in the pathogenesis of CA-VTE. The pathway includes the release of selectins, which act as the initial mediators for the recruitment of white blood cells to the vessel wall, and the exposure of vWF multimers, which form adhesive networks for the adhesion of monocytes and neutrophils to the vascular endothelium, supporting the extravasation of leukocytes and the progression of the inflammatory response. Neutrophils seem to play an important role in malignancy-related inflammation and hypercoagulation. Depletion of neutrophils has an inhibitory effect on coagulation. Neutrophil extracellular traps (NETs), composed of DNA/histone strings and immunomodulatory factors, are known to potentiate thrombus formation (through several mechanisms) and are major components of thrombi. It was demonstrated that NETs bind to vWF networks and platelets, promote fibrin generation through coagulation factor XII, and amplify TF activity [13].

Moreover, platelets and cancer-related changes in platelet function contribute to venous thrombosis in patients with cancer, and individuals who had already had high platelet counts before they developed cancer had a higher risk of CA-VTE. Several studies have shown that biomarkers of platelet activation are increased in cancer patients. For example, in patients with pancreatic cancer, elevated levels of platelet factor 4 (PF4) were found, and these elevated levels were associated with an approximately threefold increase in the risk of VTE. The role of platelets in thrombosis has also been studied in animal models of malignancies. In a model of pancreatic cancer, the antiplatelet agent clopidogrel reduced the binding of tumor-derived microvesicles to the site of thrombosis. Another study found that TF-positive tumor-derived microvesicles can activate platelets via the thrombin-dependent signaling pathway. Furthermore, cancer patients with high levels of circulating soluble P-selectin (a marker of platelet activation) have a higher risk of VTE. P-selectin is expressed by both platelets and endothelial cells. P-selectin expression by endothelial cells could aggravate VTE by recruiting leukocytes. It has been shown that inhibition of P-selectin reduces VTE in an in vitro model and that P-selectin can be responsible for the recruitment of leukocytes. In addition, cancer-derived changes influence platelet function, which could lead to neo-angiogenesis and therefore increase the risk of metastasis [14,15].

Furthermore, other general factors, such as venous stasis due to tumor mass compression, infections, and hospital-stay-related immobilization, may contribute to the increased risk of VTE in patients with oral cancer. Finally, surgery for oral cancer often involves long procedures, especially in cases of simultaneous reconstruction, leading to prolonged hospitalization and bed rest, and cytostatic drug therapy was also related to an increased risk of thrombotic events in patients with oral cancer [16,17,18]. In a retrospective study of patients with head and neck cancer, the incidence of VTE in patients who underwent reconstruction after cancer resection ranged from 1.4 to 5.8%, and the main predictors for VTE were elderly, prior thrombosis, obesity, and the need for blood transfusion [19].

**Figure 1:**
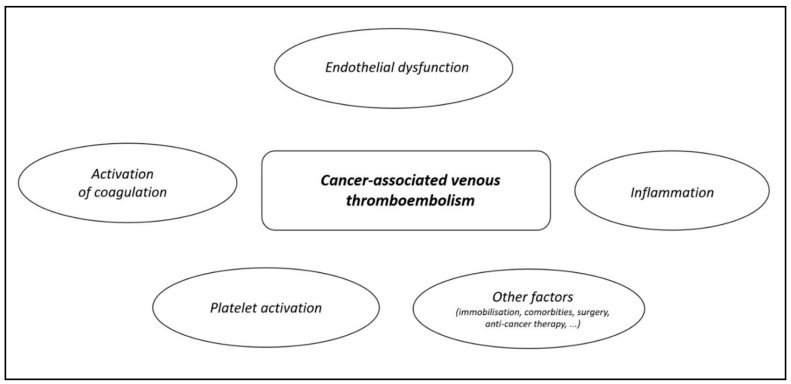
Pathophysiology of cancer-associated venous thromboembolism.

Summarizing, several of the above-mentioned factors (Figure 1) can contribute to an increased risk of CA-VTE in patients with OFC, and therefore, this issue should not be omitted in the management of these patients.

Another issue is the problem of bleeding risk, which can be, unsurprisingly, enhanced by anticoagulation therapy. The exact rate and risk factors for bleeding in patients with CA-VTE receiving anticoagulation are not well established. Several studies have tried to address this issue. In a multicenter retrospective registry enrolling 3027 consecutive patients with acute symptomatic VTE (in Japan), during a median follow-up period of 199 days, major bleeding occurred in 72 individuals. The incidence of major hemorrhage was 5.8% at 3 months, 13.8% at 1 year, 17.5% at 2 years, and 28.1% at 5 years. The most frequent type of major bleeding was gastrointestinal bleeding (47%). Terminal cancer, chronic kidney disease, and gastrointestinal cancer were identified as independent risk factors for major bleeding [20]. Another analysis of nationwide registries (in Denmark) showed that among CA-VTE patients treated with rivaroxaban, major bleeding occurred in 1.9% of patients, particularly in those with genitourinary and lung malignancies [21]. In another retrospective study in patients with CA-VTE (including 26,894 CA-VTE patients), there were 1204 bleeding events, representing a bleeding rate of 4.4% per patient-year. The rates of hemorrhage varied by cancer type, with the highest rate found in upper gastrointestinal cancers (8.6%) and the lowest in breast cancer (2.9%) [22]. Thus, the risk of bleeding should also be taken into account when choosing the appropriate therapeutic strategy for patients with CA-VTE.

## 4. Current Recommendations on Treatment of CA-VTE

Several clinical practice guidelines on the prevention and treatment of CA-VTE are currently available [23]. These include International Initiative on Thrombosis and Cancer (ITAC) clinical practice guidelines [24] published in 2019 (subsequently endorsed by the International Society on Thrombosis and Haemostasis—ISTH), American Society of Clinical Oncology (ASCO) guidelines [25] published in 2019, 2021 American Society of Hematology (ASH) guidelines [26], National Comprehensive Cancer Network (NCCN) clinical practice guidelines [27], and European Society of Medical Oncology clinical practice guidelines on venous thromboembolism in cancer patients [28] published in 2023. In addition, there is a paper discussing specifically the role of DOACs in these patients, which was published by ISTH in 2018 [29]. Summarizing the CA-VTE treatment recommendations from these guidelines, low molecular weight heparins (LMWHs), unfractionated heparin (UFH), fondaparinux, or selected DOACs (rivaroxaban, apixaban or edoxaban after at least five days of parenteral anticoagulation) are currently recommended for initial pharmacological anticoagulation. Subsequently, DOACs or LMWHs for at least six months should be preferred over vitamin K antagonists (VKA) for maintenance treatment. The selection of an anticoagulant drug should reflect the individual risk of hemorrhage and recurrent thrombosis, kidney and liver function status, in-/out-patient status, burden of laboratory testing, drug half-life and reversibility, and preference of the patient [23]. If pharmacological anticoagulation is contraindicated, retrievable inferior vena cava (IVC) filter placement should be considered. Additionally, catheter-directed thrombolysis can be used in those who are at risk of limb loss or are severely symptomatic despite anticoagulation and who have a low risk of bleeding, and systemic or catheter-derived thrombolysis or embolectomy should be considered in patients with hemodynamically unstable pulmonary embolism with a low risk of bleeding [27].

## 5. Pharmacological Treatment of CA-VTE

Heparin-based anticoagulation therapy (Table 1) represented the standard of CA-VTE therapy until 2018 and is still preferred in selected patients (especially those with gastrointestinal or genitourinary cancers) [23]. UFH is usually used in patients with severely reduced kidney function or as an initial anticoagulant (in the form of intravenous bolus administration) in patients with high-risk pulmonary embolism, as its administration is associated with several disadvantages, namely the need for frequent subcutaneous doses or continuous intravenous administration, frequent laboratory monitoring with dose adjustment, and a high risk of heparin-induced thrombocytopenia (HIT). The majority of these disadvantages can be avoided with LMWH administration, and these drugs can also be used for extended anticoagulation with acceptable results [30,31], but there is still a risk of HIT induction. Fondaparinux, a pentasaccharide-based indirect parenteral factor Xa inhibitor (FXaI), can be used for initial and also prolonged anticoagulation for CA-VTE [32], especially in those with a contraindication to heparin-based anticoagulants (such as documented HIT or heparin/LMWH allergies); however, there is no larger study examining its efficacy/safety profile in patients with CA-VTE. Nevertheless, the drug offers predictable anticoagulation, simple dosing (Table 1), no need for routine laboratory monitoring, and a very low risk of HIT development [33].

Furthermore, four recent randomized controlled clinical trials, namely HOKUSAI-VTE CANCER [34], SELECT-D [35], ADAM-VTE [36], CASTA-DIVA [37], and CARAVAGGIO [38], introduced oral FXaI-based DOACs (Table 2), edoxaban, rivaroxaban, and apixaban, to the drug treatment of CA-VTE. In addition, the CANVAS [39] pragmatic trial compared recurrent VTE, bleeding, and death in cancer patients following an initial VTE treated with either DOAC or LMWH therapy; however, the trial did not have a blinded design. Based on the design of these studies, edoxaban requires at least five days of initial parenteral anticoagulation; rivaroxaban and apixaban can be administered as initial anticoagulant therapy without the need for parenteral drug pre-treatment. In these studies [34,35,36,37,38,39], DOAC therapy compared to LMWHs (dalteparin) showed non-inferiority or superiority in efficacy (reduction of recurrent VTE), with an increased risk of major bleeding reported in the HOKUSAI-VTE CANCER and SELECT-D trials [34,35]. The risk of non-major hemorrhage was significantly higher with edoxaban in the HOKUSAI-VTE CANCER [34], rivaroxaban in the SELECT-D [35], and apixaban in the CARAVAGGIO [38] trial, and these bleeds occurred mostly in the gastrointestinal and genitourinary tracts.

These trials (except the CANVAS trial) were designed as randomized, open-label, investigator-initiated, non-inferiority trials that compared selected oral FXaI-based DOACs (with or without a short period of LMWH pretreatment) with a comparator LMWH—dalteparin. The primary efficacy outcome was recurrent VTE, and the primary safety outcome was major bleeding. The HOKUSAI-VTE CANCER [34] trial included adult patients with cancer and acute symptomatic or incidentally detected DVT (involving the popliteal, femoral, or iliac vein or the inferior vena cava) or acute symptomatic PE. Patients had to have cancer other than basal cell or squamous cell skin cancer that was active (cancer diagnosed within the previous 6 months, recurrent, regionally advanced, or metastatic cancer, cancer treated within 6 months before patient enrollment, or hematologic cancer that was not in complete remission) or had been diagnosed within the previous 2 years and was confirmed by an objective method. The SELECT-D [35] trial included patients with active cancer (solid and hematologic malignancies; defined as a diagnosed cancer other than basal cell or squamous cell skin cancer in the previous 6 months, treated cancer within the previous 6 months, recurrent or metastatic cancer, or hematologic cancer that was not in complete remission) presenting with a primary objectively confirmed VTE, including symptomatic lower-extremity proximal DVT, symptomatic PE, or incidental PE. Additionally, patients had to be at least 18 years old with a weight of at least 40 kg, have an Eastern Cooperative Oncology Group performance status of ≤2; and have adequate hematopoietic, liver, and kidney function. The ADAM-VTE [36] trial enrolled patients 18 years of age or older with confirmed active cancer, defined as any evidence of cancer on cross-sectional or positron emission tomography imaging, metastatic disease, and/or cancer-related surgery, chemotherapy, or radiation therapy within 6 months prior to enrollment and VTE. VTE was defined as acute lower or upper extremity DVT, PE, and splanchnic or cerebral vein thrombosis confirmed by an appropriate imaging method. Patients had to have a life expectancy of more than 60 days and an Eastern Cooperative Oncology Group performance status of 2 or less. Required laboratory criteria included a platelet count ≥ 50 × 10^9^/L, normal liver function tests (<3-fold the upper limit of normal), an international normalized ratio ≤ 1.6, an estimated glomerular filtration rate ≥30 mL/min using the Cockcroft-Gault formula, and a negative pregnancy test for women of childbearing potential. A similar design was also used in the CASTA-DIVA [37] trial. In the CARAVAGGIO [38] trial, patients were included if they met the following criteria: Adults with cancer (patients with confirmed cancer other than basal cell or squamous cell carcinoma of the skin, primary brain tumor, known intracerebral metastases, or acute leukemia; active cancer was defined as cancer that had been diagnosed within 6 months prior enrollment, cancer for which anticancer treatment was being given at the time of enrollment or during the 6 months before enrollment, or recurrent locally advanced or metastatic cancer; history of cancer was defined as a diagnosis of cancer made within 2 years before enrollment) who had a newly diagnosed symptomatic or incidental proximal lower limb DVT or PE. Looking at the number of patients included in the study results analysis, the HOKUSAI-VTE CANCER [34] trial analyzed data from 1046 patients, SELECT-D [35] data from 406 patients, ADAM-VTE [36] data from 287 patients, the CASTA-DIVA [37] trial from 158 patients, and the CARAVAGGIO [38] trial from 1155 patients, respectively.

Upon a closer inspection of the main findings of these trials, in the HOKUSAI-VTE CANCER [34], a primary outcome was reported in 12.8% of patients in the edoxaban group compared to 13.5% of patients in the dalteparin group (hazard ratio [HR] 0.97; 95% confidence interval [CI] 0.70 to 1.36; *p* = 0.006 for noninferiority). Recurrent VTE occurred in 7.9% of patients in the edoxaban group and in 11.3% of patients in the dalteparin group (95% CI −7.0 to 0.2). Major bleeding was reported in 6.9% of patients in the edoxaban group compared to 4.0% of patients in the dalteparin group (95% CI 0.1 to 5.6). In the SELECT-D trial [35], a total of 26 patients (from 203 patients randomly assigned to each study arm) experienced recurrent VTE (18 dalteparin-treated patients and 8 rivaroxaban-treated patients). The 6-month VTE recurrence rate was 11% (95% CI 7% to 16%) with dalteparin compared to 4% (95% CI 2% to 9%) with rivaroxaban (HR 0.43; 95% CI 0.19 to 0.99). The 6-month rate of major bleeding was 4% (95% CI 2% to 8%) for dalteparin and 6% (95% CI 3% to 11%) for rivaroxaban (HR 1.83; 95% CI 0.68 to 4.96). The ADAM-VTE [36] trial reported that major bleeding occurred in none of 145 apixaban-treated patients compared to 1.4% of 142 dalteparin-treated patients, and recurrent VTE occurred in 0.7% of apixaban-treated patients compared to 6.3% of dalteparin-treated ones. The CASTA-DIVA [37] trial showed that recurrent VTE occurred in four rivaroxaban-treated patients and in six dalteparin-treated patients (6.4% vs. 10.1%; HR 0.75; 95% CI 0.21–2.66). Major bleeding occurred in 1.4% of rivaroxaban-treated patients vs. 3.7% of dalteparin-treated patients (HR 0.36; 95% CI 0.04–3.43). There was no significant difference in the composite end-point of major or clinically relevant non-major bleeding (12.2% vs. 9.8%; HR 1.27; 95% CI 0.49–3.26). Overall, 19 rivaroxaban-treated patients (25.7%) and 20 dalteparin-treated patients (23.8%) died during the follow-up period (HR 1.05; 95% CI 0.56–1.97). Finally, in the CARAVAGGIO trial with apixaban [38], recurrent VTE was reported in 5.6% of patients in the apixaban group compared to 7.9% of patients in the dalteparin group (HR 0.63; 95% CI 0.37 to 1.07; *p* < 0.001 for noninferiority). Major hemorrhage was reported in 3.8% of apixaban-treated patients compared to 4.0% of patients treated with dalteparin (HR 0.82; 95% CI 0.40 to 1.69; *p* = 0.60).

As mentioned above, DOAC therapy for CA-VTE was also tested in a non-blinded hybrid comparative efficacy non-inferiority trial, CANVAS [39], with randomized and preference cohorts. In this trial, 671 individuals with CA-VTE were randomized between December 2016 and April 2020. In addition, 140 other individuals declined randomization and chose their preferred anticoagulant strategy. Patients were followed for 6 months. Randomized patients were assigned to DOACs or LMWHs in a 1:1 ratio. The transition to VKA was allowed in the LMWH arm of the study. The primary outcome of this study was recurrent VTE. Secondary outcomes included mortality and bleeding. The results of this study showed the non-inferiority of DOACs compared to LMWHs in terms of the primary outcome.

There is no randomized controlled clinical trial examining the efficacy/safety profile of direct oral thrombin (factor IIa) inhibitor dabigatran in patients with CA-VTE; thus, this agent should only be considered in selected cases (for example, if all oral FXaI are not tolerated or if there is a recurrence of VTE despite oral FXaI therapy), after appropriate evaluation of the risk/benefit ratio. The next question is: What is the level of evidence for these drugs in patients with OFC?

## 6. Parenteral Anticoagulation in Patients with OFC

Looking at the evidence supporting currently recommended parenteral anticoagulation therapy (UHF, LMWH, and fondaparinux) in patients with OFC, one must remark that there are only limited data regarding this issue. Firstly, at the moment, there is no study specifically examining the efficacy/safety profile of UFH and fondaparinux in this CA-VTE patient sub-population. Dalteparin therapy (as a comparator) has been evaluated in the already mentioned randomized controlled trials. In the HOKUSAI-VTE CANCER trial [34], 11.5% of dalteparin-treated patients (from a total of 524 patients) had “other type” of cancer, which included oral and facial malignancies. The sub-analysis of results in those patients with “other type” of cancer was not reported. In the SELECT-D trial [35], dalteparin (dosed 200 IU/kg daily during the first month, then 150 IU/kg daily up to six months) was administered as a rivaroxaban comparator. In this study, patients with OFC were not included (although this type of cancer was not reported as an exclusion criterion for patient enrollment). In the ADAM-VTE trial [36], only one dalteparin-treated (same dose regimen was used as in the SELECT-D trial) patient (0.7%) had ears, nose, and throat cancer (sub-type was not reported). Finally, in the CARAVAGGIO trial [38], 1.4% of patients receiving dalteparin (from a total of 579 patients) had head and neck malignancies. The sub-analysis of efficacy and safety endpoints in this patient sub-population was not reported. Additionally, there is no study specifically examining the efficacy/safety profiles of enoxaparin and nadroparin in patients with oral cancer. Therefore, although LMWHs are still preferred for initial anticoagulation in patients with CA-VTE [40], there are a lack of data regarding their efficacy and safety in patients with oral cancer, and there is definitely a need for further research in this area.

## 7. Oral anticoagulation in Patients with OFC

Unfortunately, the lack of evidence is still present when considering the data on the use of oral anticoagulation in patients with OFC and CA-VTE. In randomized studies, 17 patients with cancer localized in this area received apixaban [36,38], and some of the 48 patients with “other type of cancer” treated with edoxaban could also have OFC [34]. None of the already mentioned randomized studies published a sub-analysis of results in this patient sub-population. Furthermore, no study examined the use of vitamin K antagonists or dabigatran in patients with OFC suffering from CA-VTE. Therefore, the efficacy and safety data on oral anticoagulation (including oral FXaI) are limited to those already mentioned, and it is still not known whether the use of DOACs for CA-VTE in this type of population requires caution due to the increased risk of bleeding complications (such as in patients with gastrointestinal or genitourinary cancer) or not. Definitely, this is another area that is open for further research.

## 8. Non-Pharmacological Treatment of CA-VTE in Patients with OFC

IVC filter placement [41] and catheter-directed local thrombolysis [42] can be considered to prevent pulmonary embolism or treat DVT in CA-VTE patients who cannot receive pharmacological anticoagulation or do not respond optimally to anticoagulation. Catheter-directed (or catheter-derived ultrasound-facilitated) local thrombolysis [43] or embolectomy [44] can be considered non-pharmacological treatment in high-risk pulmonary embolism CA-VTE patients with a low risk of bleeding [27]. Brailovsky et al. [42] in their observational study analyzed 31,124 patients with cancer-associated proximal DVT, of whom 1290 (4%) received catheter-directed thrombolysis. In this analysis, there was no significant difference in in-hospital mortality among patients undergoing catheter-directed treatment compared with those treated with anticoagulation alone (2.6% versus 1.9%; *p* = 0.23). Nevertheless, catheter-based therapy was associated with an increased risk of intracranial bleeding, a higher risk of blood transfusion, and higher rates of procedure-related local bleeding complications (hematomas). The length of in-hospital stay and hospital financial costs were also higher in patients undergoing catheter-directed therapy. In another single-center retrospective cohort study, pharmaco-mechanical thrombectomy [45] achieved clinical success in 90.5% of patients with cancer-associated DVT. None of the patients undergoing catheter-based therapy suffered from procedure-related or major complications. Minor bleeding occurred in 23.8% of patients in the catheter-directed thrombectomy group compared with 10.0% of patients receiving anticoagulation alone, which was not significant. However, this study analyzed only 51 patients, of whom 21 underwent catheter-based therapy. These studies were not dedicated to patients with OFC and did not report results in this specific sub-population. Although there is no study examining the use of catheter-directed thrombolysis/embolectomy in patients with OFC and high-risk pulmonary embolism, there are several cases of its successful use in patients with other types of malignancies and high-risk cancer-associated pulmonary embolism [43,44].

## 9. How to Treat CA-VTE in Patients with OFC?

**Figure 2:**
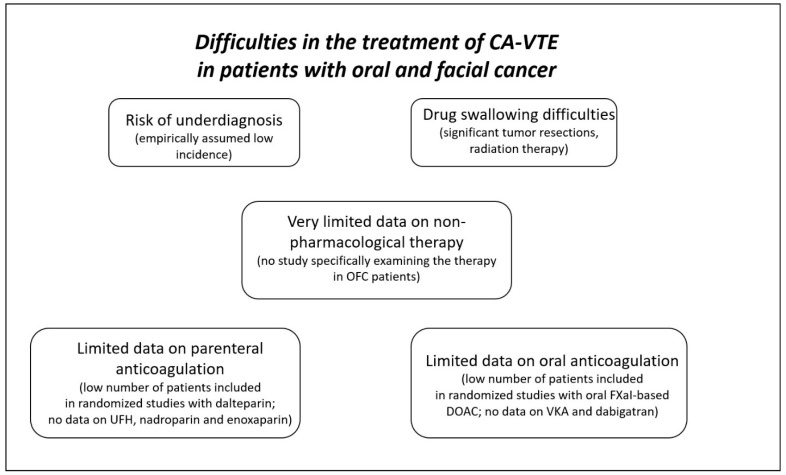
Specific problems in the management of CA-VTE in OFC patients. CA-VTE—cancer-associated venous thromboembolism; DOACs—direct oral anticoagulants; FXaI—activated factor X inhibitors; OFC—oral and facial cancer; UFH—unfractionated heparin; VKA—vitamin K antagonists.

As discussed previously, to date, there is no approach that is supported by stronger evidence from clinical studies. Therefore, this question does not have a satisfactory response. The recommendation for the general population of patients with CA-VTE should probably be applied to these patients until more data are available. As there are no reports on the increased risk of bleeding with oral FXaI, DOAC therapy or LMWH-based therapy could potentially be used as an alternative to treat CA-VTE in patients with OFC. The decision on treatment strategy should probably be based on an individual evaluation of the risk/benefit ratio. Furthermore, there might be patient sub-population difficulties with oral anticoagulation (Figure 2). For example, swallowing difficulties after significant tumor resections can limit pill ingestion. Different CA-VTE-approved DOACs have different pill profiles (Table 3), and these differences can be considered when choosing the drug for oral anticoagulation. Alternatively, rivaroxaban and edoxaban are available in the form of granules for oral suspension, which can be used, in theory, for oral anticoagulation in patients who cannot swallow the pill (although this drug form is generally not recommended in patients with CA-VTA). In patients who do not respond to pharmacological anticoagulation or cannot receive it, catheter-based therapies could probably be considered a bailout strategy. In addition, as mentioned, the bleeding risk should also be taken into account when choosing the therapeutic strategy; nevertheless, this risk seems not to be extremely high in patients with OFC (although exact data are missing). Perhaps anticoagulation should be omitted in patients with active bleeding and those after recent tumor resections. For these individuals, non-pharmacological treatment options should likely be considered. In addition, other known bleeding risk factors in CA-VTE, such as terminal cancer and chronic kidney disease, should be respected. In those patients with the risk factors mentioned above, anticoagulation therapy should probably be administered with caution, and drugs with a shorter half-life and reversal options should probably be preferred. Furthermore, extended anticoagulation therapy has little and not very well-established evidence considering CA-VTE, and this also includes patients with OFC-related CA-VTE. Definitely, more research is warranted in this area.

## 10. Conclusions

There is a lack of clinical studies dealing with the optimal treatment strategy for CA-VTE in patients with OFC. General CA-VTE treatment recommendations reflecting patient sub-population specificities should be applied, and an individual-case-consideration approach is probably needed. This area lacks satisfactory evidence and is still open for future research.

## Figures and Tables

**Table 1:** Pharmacology of parenteral anticoagulants approved for treatment of venous thromboembolism.

	Heparin (Unfractionated)	Dalteparin	Enoxaparin	Nadroparin	Fondaparinux
Target factor	IIa (indirectly—trough antithrombin III) Xa (indirectly)	Xa (indirectly)	Xa (indirectly)	Xa (indirectly)	Xa (indirectly)
Indication for treatment of DVT and PE	Yes	Yes	Yes	Yes	Yes
Way of administration	Intravenous (bolus + continuous infusion) subcutaneous (multiple daily doses needed)	subcutaneous (once or twice daily)	subcutaneous (twice daily)	subcutaneous (twice daily)	subcutaneous (once daily)
Data for CA-VTE	No	Yes (as comparator)	No	No	No
Risk of HIT	High	Low	Low	Low	Extremely low
Liver metabolism or elimination	No	No	Yes	No	No
Kidney elimination	No	Yes	Low	Yes	Yes
Half-life	One hour (intravenous) 2 h (subcutaneous)	3–4 h	3–5 h	3.5 h	17 h
Test for drug levels assessment	ACT APTT	Anti-Xa activity	Anti-Xa activity	Anti-Xa activity	Anti-Xa activity (drug-specific)

**Table 2:** Pharmacology of DOACs approved for treatment of venous thromboembolism.

	Apixaban	Dabigatran	Edoxaban	Rivaroxaban
Target factor	Xa	IIa (thrombin)	Xa	Xa
Indication for treatment of DVT and PE	Yes	Yes	Yes	Yes
Drug form	Directly-acting	Prodrug (dabigatran-etexilate)	Directly-acting	Directly-acting
Data for CA-VTE	Yes	No	Yes	Yes
Need for pre-treatment with parenteral anticoagulation	No (higher dose first 7 days recommended)	Yes	Yes	No (higher dose first 21 days recommended)
Liver metabolism	Yes (CYP3A4/5, CYP21A2, CYPSC8, CYP2C9/19, CYP2J2)	No	Yes (carboxylesterase 1, CYP3A4/5)	Yes (CYP3A4, CYP2J2)
Kidney elimination	25–30%	75–80%	45–50%	60–65%
Protein binding	87%	35%	20%	95%
Half-life	8–15 h	12–17 h	8–10 h	9–13 h
Onset of action	1–3 h	0.5–2 h	1–2 h	2–4 h
Test for drug levels assessment	Anti-Xa activity (drug-specific) LC-MS	Diluted thrombin time ecarin time LC-MS	Anti-Xa activity (drug-specific) LC-MS	Anti-Xa activity (drug-specific) LC-MS

CYP—cytochrome P450; DVT—deep vein thrombosis; LC-MS—liquid chromatography-mass spectrometry; PE—pulmonary embolism; IIa—activated factor II; Xa—activated factor X.

**Table 3:** Drug design of DOACs approved for treatment of cancer-associated venous thromboembolism.

	Apixaban	Edoxaban	Rivaroxaban *
Drug dosing	10 mg b.i.d. for first 7 days followed by 5 mg b.i.d. (2.5 mg b.i.d. for reduced dosing)	60 mg q.d. (30 mg q.d. for reduced dosing)	15 mg b.i.d. first 21 days followed by 20 mg q.d. (15 mg q.d. for reduced dosing)
Pill drug dosage	5 mg 2.5 mg	60 mg 30 mg	20 mg 15 mg
Pill design	Film-coated tablet	Film-coated tablet	Film-coated tablet
Pill shape	Oval (5 mg) Round (2.5 mg)	Round	Round biconvex
Pill dimension	9.73 x 5.16 mm (5 mg) 5.95 mm ^+^ (2.5 mg)	10.5 mm ^+^ (60 mg) 8.5 mm ^+^ (30 mg)	6 mm ^+^ 9 mm ^++^
Granules for oral suspension	No	Yes	Yes

* For original drug; may differ in different branded drugs; ^+^ diameter; ^++^ radius of curvature.

## Data Availability

Not applicable.
